# Dynamic Reference Selection-Based Self-Localization Algorithm for Drifted Underwater Acoustic Networks

**DOI:** 10.3390/s19183920

**Published:** 2019-09-11

**Authors:** Jingjie Gao, Xiaohong Shen, Haodi Mei, Zhichen Zhang

**Affiliations:** 1 School of Information Engineering, Chang’an University, Xi’an 710064, China; 2 Key Laboratory of Ocean Acoustics and Sensing, Ministry of Industry and Information Technology, Northwestern Polytechnical University, Xi’an 710072, China; xhshen@nwpu.edu.cn (X.S.); meihaodi@yeah.net (H.M.); sdhzzhzhch@mail.nwpu.edu.cn (Z.Z.); 3 School of Marine Science and Technology, Northwestern Polytechnical University, Xi’an 710072, China

**Keywords:** self-localization, drifted underwater acoustic networks, reference selection

## Abstract

Self-localization has become one of the major areas of research in drifted underwater acoustic networks (DUANs) since many applications are based on the knowledge of nodes’ positions. However, self-localization for DUANs faces two main challenges: the insufficient anchors and the varying network topology. Both affect the localization performance seriously. In this paper, we focus on these two challenges and propose a dynamic reference selection-based self-localization algorithm for DUANs (DRSL) to improve the localization performance. First, an optimal reference selection scheme is presented to solve the insufficient anchors’ problem. The selected optimal reference node can not only assist the insufficient anchors in accomplishing the localization procedure, but also obviously increase the localization accuracy. Based on the proposed optimal reference selection scheme, a dynamic reference selection-based self-localization algorithm is proposed to solve the topology changing problem. The proposed algorithm can improve the localization performance for DUANs significantly by selecting the reference node dynamically according to the predicted network topology, which is more suitable for DUANs with mobile sensor nodes. Simulation results show that the proposed DRSL algorithm can increase the localization accuracy greatly with insufficient anchor nodes and varying network topology. In addition, DRSL algorithm also has a lower communication cost than other anchor-free approaches, which distinctly demonstrates the advantages of the proposed DRSL algorithm.

## 1. Introduction

Underwater Acoustic Networks (UANs) are wireless networks defined by a collection of low-cost and resource-limited sensor nodes. Since UANs have more significant advantages than traditional wired networks including easy deployment, self-management, and large detection, it has become an important choice for a vast number of missions at sea such as data acquisition, target tracking, and remote sensing. However, influenced by the sea waves and ocean currents, nodes in UANs are often drifted, other than static, which increases the difficulty of accomplishing many network applications. Therefore, it is important and practical to do some research into drifted underwater acoustic networks (DUANs).

Self-localization has become one of the major areas of research in DUANs, since many applications such as data collection or routing protocols are based on the knowledge of nodes’ positions. Apart from the challenge that global positioning systems (GPS) is impractical due to the high attenuation of electromagnetic signals in underwater environment, self-localization for DUANs also has two main challenges: (1) topology changing caused by node’s movement (2) insufficient anchors caused by hard GPS connectivity, limited energy, and harsh underwater communication channel. Therefore, it is important and essential to design a localization algorithm for DUANs which can overcome both challenges to improve the localization performance greatly.

For UANs, there have been a lot of localization algorithms recently. Some of the existing algorithms work with the range information between each node such as dive and rise localization (DNRL), localization with directional beacons (LDB), multi-stage localization (MSL), large-scale hierarchical localization (LSHL) and AUV (Autonomous Underwater Vehicle)-aided localization (AAL) methods. These methods work well in static networks, but are not appropriate for DUANs with drifted nodes. Some methods need AUV to assist which will increase the localization cost dramatically [1,2,3,4,5,6,7,8,9,10,11,12,13,14,15,16]. The range-free algorithms based on Sequential Monte Carlo (SMC) method can provide simple and low-cost approaches to estimate the node’s position [17,18,19]. However, these algorithms are not as accurate as the range-based algorithms and need more anchors to characterize the distribution of node’s location. For mobile underwater networks, inertial navigation systems (INS) can overcome the problem of mobility and no GPS, which however, will increase the cost of localization system and introduce large error accumulation [20,21,22,23,24,25]. SLMP (scalable localization with mobility prediction for underwater sensor networks) algorithm proposed in [26] and HLA (a hybrid localization algorithm for mobile underwater acoustic networks) in [27] work well in mobile networks. However, both SLMP algorithm and HLA algorithm need a lot of anchor nodes which are not practical in real conditions. Some anchor-free algorithms have been proposed which do not need any anchor assistance to obtain the unknown nodes’ positions such as motion-aware self-localization (MASL), anchor-free localization (AFL) and collaborative localization (CL) methods. However, the major drawbacks of anchor-free localization algorithms are the high communication cost and the low accuracy [28,29,30].

In this paper, we focus on the problem of accurate self-localization with insufficient anchors and varying topology for DUANs. For this problem, we propose a dynamic reference selection-based self-localization algorithm for DUANs. First, we propose an optimal reference selection scheme to solve the insufficient anchors’ problem. The optimal reference node can assist anchors in accomplishing the localization procedure with high localization accuracy. Secondly, we propose a dynamic reference selection-based self-localization algorithm by combining the prediction-based localization algorithm with the proposed optimal reference selection scheme. The algorithm can select the reference node dynamically according to the network’s predicted topology and mobility pattern which is more suitable for DUANs with mobile sensor nodes.

The rest of this paper is organized as follows. In Section 2, we will describe the network model including the network architecture, the mobility model, and the time model. Then, in Section 3, we will describe the proposed dynamic reference selection-based self-localization algorithm for DUANs in details. Following that, we will analyze the simulation results of the proposed algorithm in Section 4. Finally, we conclude the paper in Section 5.

## 2. Network Model

Since nodes in DUANs always have different positions over time due to the drifted movement by sea waves, in this paper, we consider a time varying network model to represent DUANs which is denoted as $G^{k}=\left\{V^{k},L^{k}\right\}$. $V^{k}=\left\{\Phi^{k},\Gamma^{k}\right\}$ is the collection of all anchor nodes $\Phi^{k}$ and all ordinary nodes $\Gamma^{k}$ at time *k*. $L^{k}=\left\{\left(i,j\right)|i,j\in V^{k}\right\}$ is the set of communication links between each node in the network. In DUANs, if $d_{ij}\leq R_{c}$, node *i*, and node *j* can communicate with each other, where $d_{ij}$ is the distance between node *i* and *j*, $R_{c}$ is the communication range. In this paper, we assume that all nodes in the network can communicate with each other.

### 2.1. Network Architecture

In this paper, there are 2 kinds of nodes including anchors and ordinary nodes.

(1) Anchors are nodes which positions are accurately known before the localization process;

(2) Ordinary nodes are nodes which should get their positions by communicating with anchors directly or indirectly. Ordinary nodes can be divided into 2 types: the reference nodes and the unknown ordinary nodes. Reference nodes are nodes which are selected by anchors to assist in the localization process, while unknown ordinary nodes are nodes which should get their positions by communicating with anchors and selected reference nodes.

**Figure 1:**
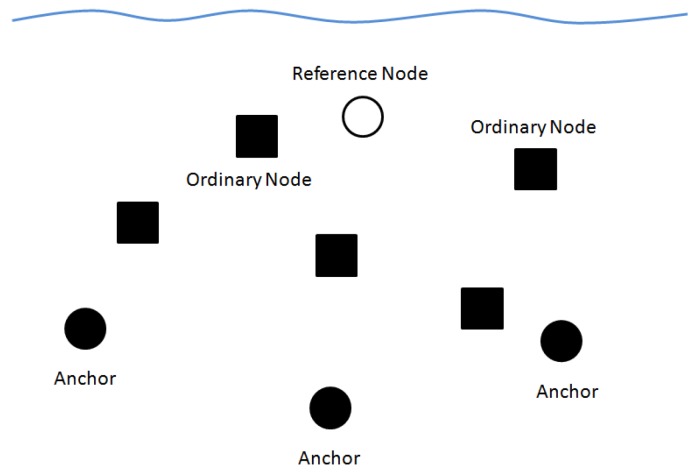
The network architecture.

Figure 1 shows the network architecture. In this paper, we assume that only one reference node is needed for localization. Other conditions can be extended from this assumption.

### 2.2. The Mobility Model

**Figure 2:**
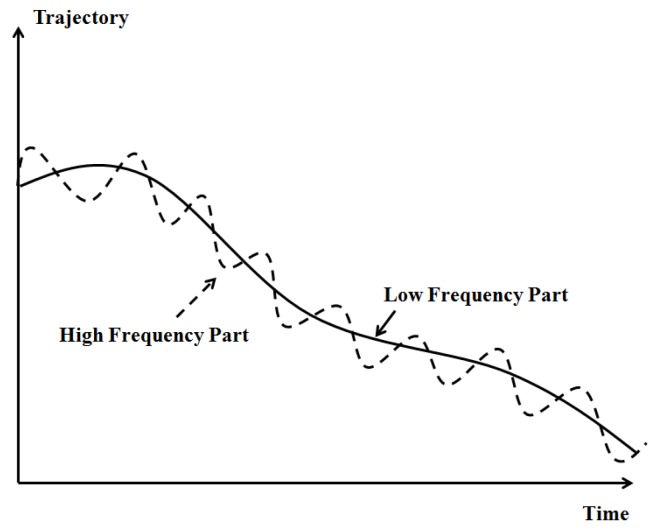
The mobility model of drifted node.

Nodes in DUANs are always moving continuously with water currents and sea waves etc. The mobility pattern of drifted node in DUANs is a complex but not totally random process. The pattern is in fact a 6 DOF (Degree of Freedom) motion including pitch, roll, yaw, pitch, roll, and heave. In addition, the mobility pattern also contains 2 parts, the low frequency part and the high frequency part. The low frequency part is the main portion of the movement caused by the second order wave force. The high frequency part is a reciprocating motion created by the first order wave force. The drifted movement is an overlay of these 2 motion parts as can be seen in Figure 2.

Since the high frequency part is a reciprocating motion which is not the main portion for DUANs, in this paper, we only focus on the low frequency movement part which indicates the primary trajectory of the drifted node.

### 2.3. The Time Model

Nodes in DUANs are always floating and mobile as time varies. Therefore, we should define a time model to indicate the mobility pattern. The time model designed in this paper comprises 3 different kinds.

- Sampling period $t_{0}$: sampling period $t_{0}$ is the basic time unit during the localization process, nodes in the networks will communicate with each other during each $t_{0}$;
  $$t_{0}=N\times \frac{1}{f}$$
  where *f* is the frequency of high frequency mobility pattern described in the last section, *N* is a proper sampling ratio.
- Estimation period $T_{0}$: Estimation period $T_{0}$ is comprised by some $t_{0}$ which is used for the mobility pattern estimation.
  $$T_{0}=N_{0}t_{0}$$
- Test period $T_{s}$: $T_{s}$ is used for re-estimation procedure to eliminate the error accumulation.

**Figure 3:**
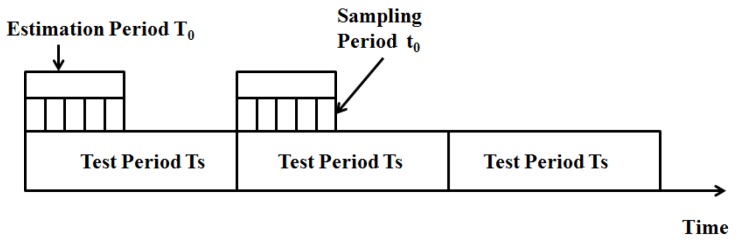
The time model of DUANs.

The time model is as shown in Figure 3.

## 3. Localization Algorithm

In this section, we will present a dynamic reference selection-based self-localization algorithm for DUANs (DRSL). The proposed algorithm is elaborated from 3 aspects: (1) The optimal reference selection scheme; (2) The dynamic reference selection-based self-localization algorithm; (3) The implementation of DRSL algorithm. Now, we will describe the three aspects in detail.

### 3.1. The Optimal Reference Selection Scheme

As we know, in three-dimensional underwater networks, ordinary node should communicate with at least four anchors to get its position. However, due to the anchor node failure or the harsh underwater communication environment, it is impossible to satisfy the demand of 4-anchor connectivity in DUANs all the time. Therefore, it is critical to develop an optimal reference selection scheme to support insufficient anchors in accomplishing the self-localization process precisely.

In this section, an optimal reference selection scheme is proposed to increase the localization accuracy for DUANs with insufficient anchors. The scheme is also the foundation and the initialization of the dynamic reference selection-based self-localization algorithm illustrated in the next section.

Assume that there are only three anchors in the network at time *k*, $\left(x_{1}\left(k\right),y_{1}\left(k\right),z_{1}\left(k\right)\right)$, $\left(x_{2}\left(k\right),y_{2}\left(k\right),z_{2}\left(k\right)\right)$ and $\left(x_{3}\left(k\right),y_{3}\left(k\right),z_{3}\left(k\right)\right)$ which are not sufficient for the localization process conventionally. Therefore, we need to select an extra reference node $\left(x_{i}\left(k\right),y_{i}\left(k\right),z_{i}\left(k\right)\right)$ to assist the localization procedure.

**Figure 4:**
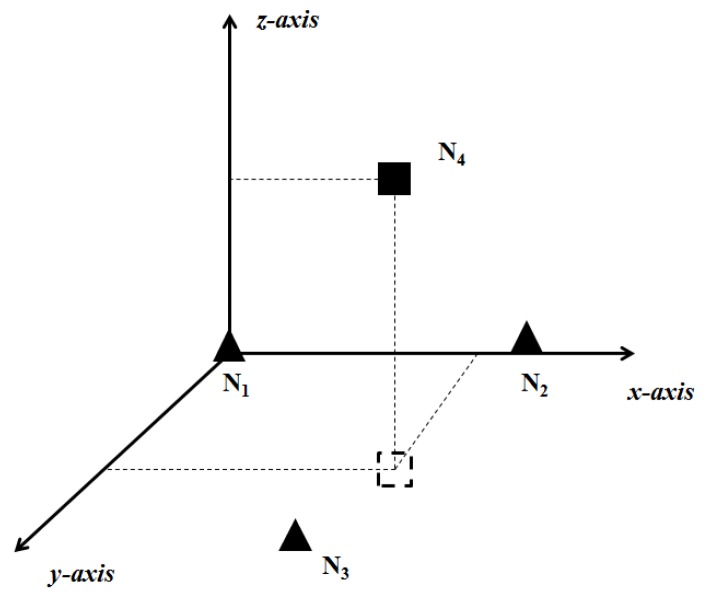
The relative coordinate system.

First, we set up a relative coordinate system as shown in Figure 4. In this figure, $N_{1}$, $N_{2}$ and $N_{3}$ are the anchor nodes respectively, while $N_{i}$ is the selected reference node. Let $N_{1}$$N_{2}$ make up the x-axis. $N_{1}$, $N_{2}$ and $N_{3}$ are in the xy plane. The z-coordinate of node $N_{i}$ is positive. Thus, the relative coordinates of the three anchors are (0,0,0), $\left({\hat{x}}_{2}\left(k\right),0,0\right)$ and $\left({\hat{x}}_{3}\left(k\right),{\hat{y}}_{3}\left(k\right),0\right)$ respectively. ${\hat{x}}_{2}\left(k\right)$,${\hat{x}}_{3}\left(k\right)$ and ${\hat{y}}_{3}\left(k\right)$ are expressed as Equation (1) to Equation (3), where θ is the rotation angle between the relative coordinate system and the geodetic coordinate system.

(1) $${\hat{x}}_{2}\left(k\right)=\sqrt{{\left(x_{2}\left(k\right)-x_{1}\left(k\right)\right)}^{2}+{\left(y_{2}\left(k\right)-y_{1}\left(k\right)\right)}^{2}+{\left(z_{2}\left(k\right)-z_{1}\left(k\right)\right)}^{2}}$$

(2) $${\hat{x}}_{3}\left(k\right)=cos\left(\theta \right)\sqrt{{\left(x_{3}\left(k\right)-x_{1}\left(k\right)\right)}^{2}+{\left(y_{3}\left(k\right)-y_{1}\left(k\right)\right)}^{2}+{\left(z_{3}\left(k\right)-z_{1}\left(k\right)\right)}^{2}}$$

(3) $${\hat{y}}_{3}\left(k\right)=sin\left(\theta \right)\sqrt{{\left(x_{3}\left(k\right)-x_{1}\left(k\right)\right)}^{2}+{\left(y_{3}\left(k\right)-y_{1}\left(k\right)\right)}^{2}+{\left(z_{3}\left(k\right)-z_{1}\left(k\right)\right)}^{2}}$$

Secondly, as mentioned in [4], we can use the least square method to solve the self-localization problem. Therefore, in this paper, the least square error is adopted to get the localization error as mentioned in Equation (4).

(4) $$E_{ij}\left(k\right)={b_{ij}\left(k\right)}^{T}\left(I-A(k){\left({A(k)}^{T}A(k)\right)}^{-1}{A(k)}^{T}\right)b_{\mathrm{ij}}\left(k\right)$$

$E_{ij}\left(k\right)$ is the least square error of unknown ordinary node *j* at time *k* when node *i* is selected as the reference node.

$$A(k)=\left[\begin{array}{ccc} 2(-{\hat{x}}_{i}\left(k\right)) & 2(-{\hat{y}}_{i}\left(k\right)) & 2(-{\hat{z}}_{i}\left(k\right))\\ 2({\hat{x}}_{2}\left(k\right)-{\hat{x}}_{i}\left(k\right)) & 2(-{\hat{y}}_{i}\left(k\right)) & 2(-{\hat{z}}_{i}\left(k\right))\\ 2({\hat{x}}_{2}\left(k\right)-{\hat{x}}_{i}\left(k\right)) & 2({\hat{y}}_{2}\left(k\right)-{\hat{y}}_{i}\left(k\right)) & 2(-{\hat{z}}_{i}\left(k\right)) \end{array}\right]$$

$$b_{ij}\left(k\right)=\left[\begin{array}{c} -{{\hat{x}}_{i}\left(k\right)}^{2}-{{\hat{y}}_{i}\left(k\right)}^{2}-{{\hat{z}}_{i}\left(k\right)}^{2}+{d_{i}^{j}\left(k\right)}^{2}-{d_{1}^{j}\left(k\right)}^{2}\\ {{\hat{x}}_{2}\left(k\right)}^{2}-{{\hat{x}}_{i}\left(k\right)}^{2}-{{\hat{y}}_{i}\left(k\right)}^{2}-{{\hat{z}}_{i}\left(k\right)}^{2}+{d_{i}^{j}\left(k\right)}^{2}-{d_{2}^{j}\left(k\right)}^{2}\\ {{\hat{x}}_{3}\left(k\right)}^{2}-{{\hat{x}}_{i}\left(k\right)}^{2}+{{\hat{y}}_{3}\left(k\right)}^{2}-{{\hat{y}}_{i}\left(k\right)}^{2}-{{\hat{z}}_{i}\left(k\right)}^{2}+{d_{i}^{i}\left(k\right)}^{2}-{d_{3}^{i}\left(k\right)}^{2} \end{array}\right]$$

$d_{1}^{j}\left(k\right),d_{2}^{j}\left(k\right),d_{3}^{j}\left(k\right)$ are the distances between the unknown ordinary node *j* and the anchor nodes at time *k*. $d_{i}^{j}\left(k\right)$ is the distance between the unknown node *j* and the selected reference node *i* at time *k*.

The aim of reference selection scheme proposed in this section is to accomplish the localization process in DUANs with insufficient anchor nodes and to improve the localization accuracy simultaneously. Therefore, the optimal reference node is selected by minimizing the global localization error in the whole network according to Equation (5).

Assume that there are *M* ordinary nodes in the network including one reference node which will be selected and M−1 unknown ordinary node. The set of ordinary nodes in relative coordinate system is expressed as $\hat{\Gamma }\left(k\right)$, while ${\hat{\Gamma }}_{i}\left(k\right)$ is the selected reference node *i*. Thus, the cost function of the optimal reference selection scheme is expressed as:

(5) $$\begin{array}{cc} \min_{{\hat{\Gamma }}_{i}\left(k\right)\in \hat{\Gamma }\left(k\right)}\;\sum_{j=1}^{i-1} & \sum_{j=i+1}^{M}{b_{ij}\left(k\right)}^{T}\left(I-A\left(k\right){\left({A(k)}^{T}A\left(k\right)\right)}^{-1}{A(k)}^{T}\right)b_{ij}\left(k\right)\\ s.t.\;A(k)= & \left[\begin{array}{ccc} -{\hat{x}}_{i}\left(k\right) & -{\hat{y}}_{i}\left(k\right) & -{\hat{z}}_{i}\left(k\right)\\ 2({\hat{x}}_{2}\left(k\right)-{\hat{x}}_{i}\left(k\right)) & -2{\hat{y}}_{i}\left(k\right) & -2{\hat{z}}_{i}^{(}k)\\ 2({\hat{x}}_{3}\left(k\right)-{\hat{x}}_{i}\left(k\right)) & 2({\hat{y}}_{3}\left(k\right)-{\hat{y}}_{i}^{(}k)) & -2{\hat{z}}_{i}\left(k\right) \end{array}\right]\\ b_{ij}\left(k\right)= & \left[\begin{array}{c} -{\left({\hat{x}}_{i}\left(k\right)\right)}^{2}-{\left(y_{i}\left(k\right)\right)}^{2}-{\left({\hat{z}}_{i}\left(k\right)\right)}^{2}+\Delta d_{1}^{j}\left(k\right)\\ {\hat{x}}_{2}{\left(k\right)}^{2}-{\left({\hat{x}}_{i}\left(k\right)\right)}^{2}-{\left({\hat{y}}_{i}\left(k\right)\right)}^{2}-{\left({\hat{z}}_{i}\left(k\right)\right)}^{2}+\Delta d_{2}^{j}\left(k\right)\\ {\hat{x}}_{3}{\left(k\right)}^{2}-{\left({\hat{x}}_{i}^{(}k)\right)}^{2}+{\hat{y}}_{3}{\left(k\right)}^{2}-{\left({\hat{y}}_{i}^{(}k)\right)}^{2}-{\left({\hat{z}}_{i}^{(}k)\right)}^{2}+\Delta d_{3}^{j}\left(k\right) \end{array}\right]\\ \Delta d_{n}^{j}= & d_{j}^{2}-d_{n}^{2}\;n=1,2,3\\ {\hat{\Gamma }}_{i}\left(k\right)= & \left[\begin{array}{c} {\hat{x}}_{i}\left(k\right)\\ {\hat{y}}_{i}\left(k\right)\\ {\hat{z}}_{i}\left(k\right) \end{array}\right] \end{array}$$

### 3.2. The Dynamic Reference Selection-Based Self-Localization Algorithm

In this section, we will propose a dynamic reference selection-based self-localization algorithm for DUANs. The algorithm combines the optimal reference selection scheme and the prediction-based self-localization algorithm together to select the optimal reference node dynamically. The dynamic reference selection-based self-localization algorithm can solve both the insufficient anchors’ problem and the topology changing problem which is more appropriate for DUANs with mobile sensor nodes and can increase the localization accuracy dramatically.

#### 3.2.1. The Prediction-Based Self-Localization Algorithm

Considering the mobility model and the observation model of DUANs, we apply the extension Kalman filter (EKF) method to predict the node’s position and the mobility pattern in DUANs [31,32].

Mobility model:

(6) $$\begin{array}{cc} {\hat{\Gamma }}_{j}\left(k+1\right) & =\left[\begin{array}{c} X_{j}\left(k+1\right)\\ v_{j}\left(k+1\right)\\ a_{j}\left(k+1\right) \end{array}\right]=F{\hat{\Gamma }}_{j}\left(k\right)+\omega_{j}\left(k\right) \end{array}$$

where $v_{j}\left(k+1\right)$ is the velocity of node *j* at time k+1, $a_{j}\left(k+1\right)$ is the acceleration of node *j* at time k+1, *F* is the state transformation matrix which can be expressed as

F=100T0(−1+αt0+e−αT0)(−1+αt0+e−αT0)α2α2010T0(−1+αt0+e−αT0)(−1+αt0+e−αT0)α2α2001T0(−1+αt0+e−αT0)(−1+αt0+e−αT0)α2α20001(1−e−αt0)(1−e−αt0)αα0000e−αt0

$$\omega_{j}\left(k\right)=\left[\begin{array}{c} \omega_{1}\left(k\right)\\ \omega_{2}\left(k\right)\\ \omega_{3}\left(k\right)\\ \omega_{4}\left(k\right)\\ \omega_{5}\left(k\right) \end{array}\right]$$

$$X_{j}\left(k+1\right)=\left[\begin{array}{c} x_{j}\left(k+1\right)\\ y_{j}\left(k+1\right)\\ z_{j}\left(k+1\right) \end{array}\right]$$

where $\omega_{j}\left(k\right)$ is the mobility noise vector, α is the model factor. $t_{0}$ is the sampling period.

Observation model:

The observation model is derived from the range measurement as shown in Equations (7) and (8).

(7) $$\begin{array}{cc} Z_{j}\left(k+1\right) & =\left[\begin{array}{c} Z_{\left(1,j\right)}\left(k+1\right)\\ Z_{\left(2,j\right)}\left(k+1\right)\\ Z_{\left(3,j\right)}\left(k+1\right)\\ Z_{\left(i,j\right)}\left(k+1\right) \end{array}\right] \end{array}$$

(8) $$\begin{array}{cc} Z_{\left(l,j\right)}\left(k+1\right) & =h_{\left(l,j\right)}\left(X_{l}\left(k+1\right),X_{j}\left(k+1\right)\right)+\mu_{\left(l,j\right)}\left(k+1\right)\\ & =\|X_{l}\left(k+1\right)-X_{j}\left(k+1\right)\|+\mu_{\left(l,j\right)}\left(k+1\right),l=1,2,3,i \end{array}$$

where $Z_{\left(l,j\right)}\left(k+1\right)$ is the range measurement between node *l* and node *j* at time k+1. $\mu_{\left(l,j\right)}\left(k+1\right)$ is the noise vector, $\mu_{\left(l,j\right)}\left(k+1\right)∼N\left(0,R\left(k\right)\right)$, R(k) is the variance of observation noise.

Since the nonlinearity of the observation model as in Equation (8), we use EKF method to predict the mobile node’s position and the mobility parameter simultaneously.

$${\hat{\Gamma }}_{j}\left(k+1|k\right)=F{\hat{\Gamma }}_{j}\left(k|k\right)$$

$$P\left(k+1|k+1\right)=FP\left(k|k\right)F^{T}+Q\left(k+1\right)$$

(9) $$K\left(k+1\right)=P\left(k+1|k\right)J_{j}^{T}\left[J_{j}P\left(k+1|k\right)J_{j}^{T}+R\left(k+1\right)\right]$$

$${\hat{\Gamma }}_{j}\left(k+1|k+1\right)={\hat{\Gamma }}_{j}\left(k+1|k\right)+K\left(k\right)\left[Z\left(k\right)-h\left({\hat{\Gamma }}_{j}\left(k+1|k\right)\right)\right]$$

$$P\left(k+1|k+1\right)=P\left(k+1|k\right)-K\left(k\right)\left[J_{j}P\left(k\right)|kJ^{T}+R\left(k+1\right)\right]K^{T}\left(k+1\right)$$

where *P* is the state covariance matrix, *F* is the state transformation matrix, *Q* is the state transformation covariance matrix, *R* is the observation noise covariance matrix and $J_{\left(l,j\right)}$ is the Jacobians of the nonlinear function $h_{\left(l,j\right)}$.

(10) $$\begin{array}{cc} J_{j}= & =\left[\begin{array}{c} J_{\left(1,j\right)}\left(k+1\right)\\ J_{\left(2,j\right)}\left(k+1\right)\\ J_{\left(3,j\right)}\left(k+1\right)\\ J_{\left(i,j\right)}\left(k+1\right) \end{array}\right] \end{array}$$

(11) $$\begin{array}{cc} J_{\left(l,j\right)} & =\frac{\partial h^{\left(l,j\right)}\left(X_{l}\left(k+1\right),X_{j}\left(k+1\right)\right)}{\partial X_{l}\left(k+1\right)}\\ & =\left[\begin{array}{ccc} \frac{x_{l}\left(k+1\right)-x_{j}\left(k+1\right)}{d_{\left(l,j\right)}\left(k+1\right)} & \frac{y_{l}\left(k+1\right)-y_{j}\left(k+1\right)}{d_{\left(l,j\right)}\left(k+1\right)} & \frac{z_{l}\left(k+1\right)-z_{j}\left(k+1\right)}{d_{\left(l,j\right)}\left(k+1\right)} \end{array}\right],l=1,2,3,i \end{array}$$

#### 3.2.2. The Dynamic Reference Selection Scheme

Based on the prediction-based self-localization algorithm, we can predict the mobility pattern of any mobile node in DUANs. According to the predicted mobility pattern, we can estimate the node’s position directly.

(12) $$\begin{array}{cc} {\hat{\Gamma }}_{j}\left(k+1|k\right) & =F\times \left[\begin{array}{c} X_{j}\left(k\right)\\ v_{j}\left(k\right)\\ a_{j}\left(k\right) \end{array}\right] \end{array}$$

According to Equations (12) and (5), we can get the dynamic reference selection scheme expressed as (13).

(13) $$\begin{array}{c} \min_{{\hat{\Gamma }}_{i}\left(k+1|k\right)\in \hat{\Gamma }\left(k+1|k\right)}\;\sum_{j=1}^{i-1}\sum_{j=i+1}^{M}{b_{ij}\left(k\;+\;1\;|\;k\right)}^{T}\left(I\;-A\left(k\;+\;1\;|\;k\right){\left({A(k\;+\;1\;|\;k)}^{T}A\left(k\;+\;1\;|\;k\right)\right)}^{-1}{A(k\;+\;1\;|\;k)}^{T}\right)b_{ij}\left(k\;+\;1\;|\;k\right)\\ \begin{array}{ccc} s.t. & A(k\;+\;1\;|\;k) & =\left[\begin{array}{ccc} -{\hat{x}}_{i}\left(k\;+\;1\;|\;k\right) & -{\hat{y}}_{i}\left(k\;+\;1\;|\;k\right) & -{\hat{z}}_{i}\left(k\;+\;1\;|\;k\right)\\ 2({\hat{x}}_{2}\left(k\;+\;1\right)-{\hat{x}}_{i}\left(k\;+\;1\;|\;k\right)) & -2{\hat{y}}_{i}\left(k\;+\;1\;|\;k\right) & -2{\hat{z}}_{i}\left(k\;+\;1\;|\;k\right)\\ 2({\hat{x}}_{3}\left(k\;+\;1\right)-{\hat{x}}_{i}\left(k\;+\;1\;|\;k\right)) & 2({\hat{y}}_{3}\left(k\;+\;1\right)-{\hat{y}}_{i}\left(k\;+\;1\;|\;k\right)) & -2{\hat{z}}_{i}\left(k\;+\;1\;|\;k\right) \end{array}\right]\\ & b_{ij}\left(k\;+\;1\;|\;k\right) & =\left[\begin{array}{c} -{\left({\hat{x}}_{i}\left(k\;+\;1\;|\;k\right)\right)}^{2}-{\left(y_{i}\left(k\;+\;1\;|\;k\right)\right)}^{2}-{\left({\hat{z}}_{i}\left(k\;+\;1\;|\;k\right)\right)}^{2}+\Delta d_{1}^{i}\left(k\;+\;1\right)\\ {\hat{x}}_{2}{\left(k\;+\;1\right)}^{2}-{\left({\hat{x}}_{i}\left(k\;+\;1\;|\;k\right)\right)}^{2}-{\left({\hat{y}}_{i}\left(k\;+\;1\;|\;k\right)\right)}^{2}-{\left({\hat{z}}_{i}\left(k\;+\;1\;|\;k\right)\right)}^{2}+\Delta d_{2}^{j}\left(k\;+\;1\right)\\ {\hat{x}}_{3}{\left(k\;+\;1\right)}^{2}-{\left({\hat{x}}_{i}\left(k\;+\;1\;|\;k\right)\right)}^{2}+{\hat{y}}_{3}{\left(k\;+\;1\right)}^{2}-{\left({\hat{y}}_{i}\left(k\;+\;1\;|\;k\right)\right)}^{2}-{\left({\hat{z}}_{i}\left(k\;+\;1\;|\;k\right)\right)}^{2}+\Delta d_{3}^{i}\left(k\;+\;1\right) \end{array}\right]\\ & {\hat{\Gamma }}_{i}\left(k\;+\;1\;|\;k\right) & =F{\hat{\Gamma }}_{i}\left(k\;|\;k\right)=\left[\begin{array}{c} {\hat{X}}_{i}\left(k\;+\;1\;|\;k\right)\\ {\hat{v}}_{i}\left(k\;+\;1\;|\;k\right)\\ {\hat{a}}_{i}\left(k\;+\;1\;|\;k\right) \end{array}\right]\\ & \Delta d_{n}^{i} & =d_{ij}^{2}-d_{nj}^{2}\;n=1,2,3\\ & d_{lj} & =\|X_{l}\left(k\;+\;1\;|\;k\right)-X_{j}\left(k\;+\;1\;|\;k\right)\|\;l=1,2,3,i \end{array} \end{array}$$

Combining the dynamic reference selection scheme and the prediction-based self-localization algorithm, we can get the dynamic reference selection-based self-localization algorithm. The self-localization procedure is shown as follows:

1. Anchors select the initial optimal reference node according to Equation (5);
2. Each unknown ordinary node predicts its own mobility pattern and position according to the range measurements as in Equation (9) and sends them to the anchors;
3. Anchors receive the predicted mobility patterns and positions obtained above, set them to be the prior information and then update the reference selection dynamically according to the predicted information as in Equation (13);
4. If the reference selection remains unchanged, the previous predicted mobility patterns will still be used to estimate the drifted nodes’ positions continually. If the reference selection changes, we will re-estimate the mobility patterns and re-predict the drifted nodes’ positions again according to Equation (9).

As described above, we can see that the dynamic reference selection-based self-localization algorithm can increase the localization accuracy with both insufficient anchors and varying topology which is more appropriate for the drifted underwater acoustic networks.

### 3.3. The Implementation of DRSL Algorithm

In this section, we will illustrate the implementation of DRSL algorithm in detail.

Settings:

(1) We apply TDMA (Time Division Multiple Address) protocol to reduce the transmission interference in DUANs during the localization process.

(2) We assume that nodes in the network have been well synchronized, so that TOA (Time of Arrival) ranging method can be well adopted.

(3) There are *M* ordinary nodes and 3 anchor nodes in the network which is not sufficient for the localization procedure.

(4) Each node has a unique ID and can communicate with each other.

The implementation procedure is as follows:

- Step 1: Each node in the network broadcasts “hello” message with the sending time stamp one by one to trigger the localization procedure;
- Step 2: After receiving the “Hello” message, each ordinary node calculates the distance between each other according to TOA ranging method;
- Step 3: Based on the distance information obtained in step 2, each ordinary node can calculate its least square localization error according to Equation (4) and then send the localization error to anchor 1;
- Step 4: Based on the receiving least square error, anchor 1 selects an initial optimal reference node according to Equation (5), and then broadcasts the initial optimal reference node’s ID to the network;
- Step 5: Based on the selected optimal reference node’s information, other unknown ordinary nodes in the network predict their mobility patterns and mobile positions according to Equations (9) and (12);
- Step 6: After getting the predicted position results of the network, anchor 1 updates the reference selection dynamically as shown in Equation (13), and then sends the update information to the network;
- Step 7: The network changes the anchor-reference group dynamically to adapt to the varying topology of DUANs.
- Step 8: If the reference selection remains unchanged, the previous predicted mobility patterns will still be used to estimate the drifted nodes’ positions continually. If the reference selection changes, we will re-estimate the mobility patterns and re-predict the drifted nodes’ positions again according to Equations (9) and (12).

**Figure 5:**
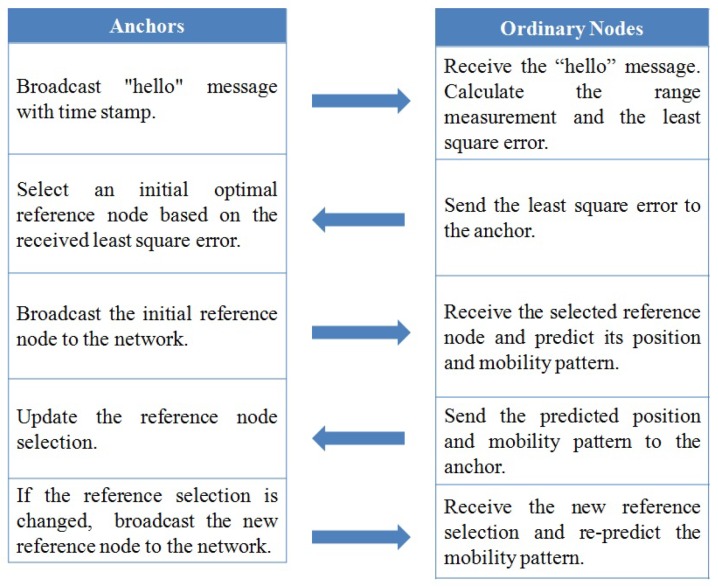
The implementation of DRSL algorithm.

The implementation of DRSL algorithm can be described as in Figure 5.

## 4. Simulation Results

In this section, simulation experiments are carried out to evaluate the performance of the proposed DRSL algorithm.

### 4.1. Simulation Settings

Ten nodes are deployed in a 1000 m × 1000 m × 1000 m region including 3 anchors and 7 unknown ordinary sensor nodes. The anchors’ positions are known and accurate before the localization process. The estimation period $T_{0}=100t_{0}$ in the simulation experiments.

Our key performance metrics in the simulation experiments are the localization error and the communication cost, respectively. The localization error is evaluated as in Equation (14). The communication cost represents the transmission data size that is needed for the localization procedure.

(14) $$Localization\;Error=\sum_{k=1}^{T}\sum_{i=1}^{N}\left\|{\hat{X}}_{i}^{k}-X_{i}^{k}\right\|$$

where ${\hat{X}}_{i}^{k}$ is the estimated position of node *i* at time *k*. $X_{i}^{k}$ is the actual position of node *i* at time *k*. *T* is the total time duration of the localization procedure; *N* is the number of unknown ordinary nodes in DUANs.

### 4.2. Results and Analysis

#### 4.2.1. The Reference Selection Analysis

**Figure 6:**
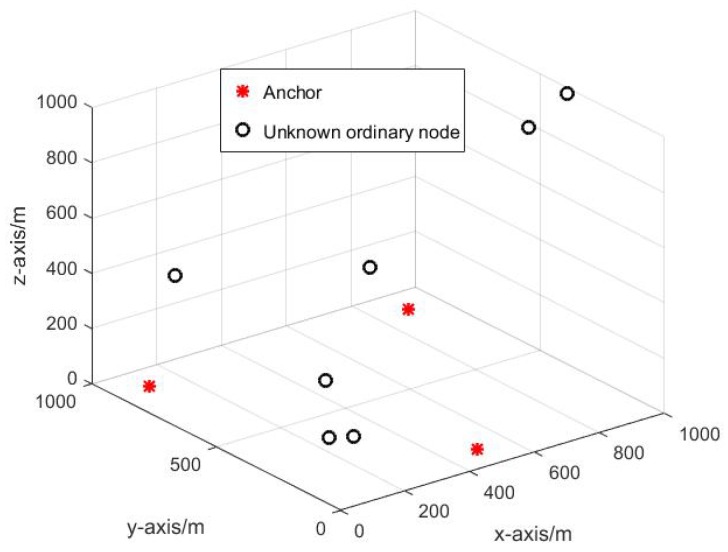
The initial topology of DUANs: red star is the anchor; black circle is the unknown ordinary node.

In this paper, several Monte Carlo experiments are performed to evaluate the performance of the proposed DRSL algorithm. Different initial network topology is adopted for different experiment. Figure 6 shows one of the network’s initial structure. As can be seen in Figure 6, because of some device failure problems, sometimes there are only 3 anchors available for the localization procedure which is insufficient obviously. Therefore, it is important to select an optimal reference node to assist the anchors in accomplishing the localization procedure precisely.

**Figure 7:**
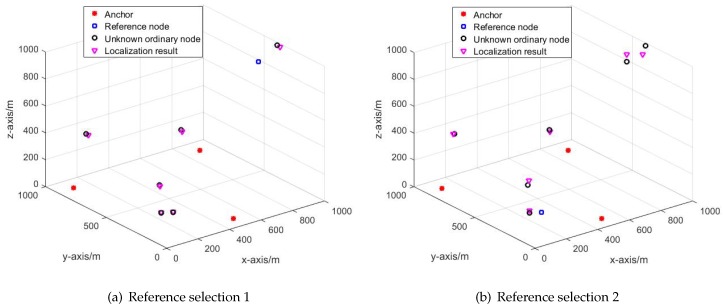
The Localization result of different reference selection.

Different selection will cause different localization result. As can be seen in Figure 7.

In Figure 7a,b have the same network structure; however, Figure 7a has a higher localization accuracy than that of Figure 7b. The only reason that causes the difference in localization accuracy is the different reference selection scheme.

**Figure 8:**
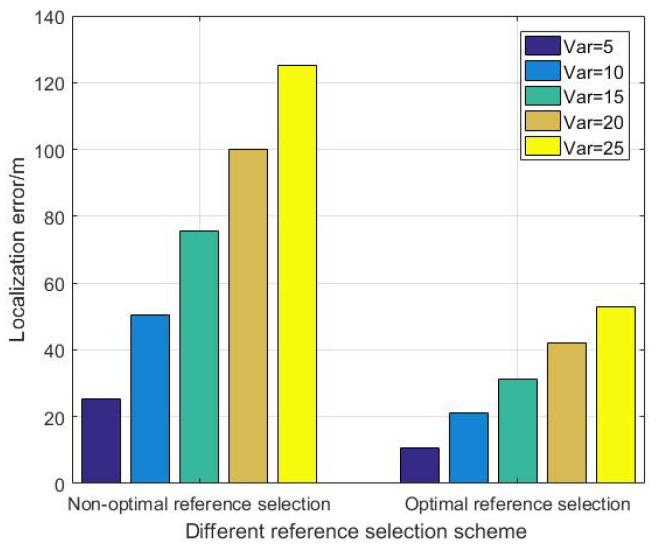
The comparison of localization error between different reference selection scheme.

Figure 8 shows the comparison of localization error at the initial time between non-optimal reference selection scheme and the proposed optimal reference selection scheme. Var is the variance of the observation error.

As it can be seen in Figure 8, the localization error increases with the growth of the observation error. Meanwhile, compared with the non-optimal reference selection scheme, the proposed optimal reference selection scheme can reduce the localization error of DUANs effectively.

Since the topology of DUANs is changing over time, the dynamic reference selection scheme proposed in this paper is more appropriate for DUANs with mobile sensor nodes. The localization performance of dynamic reference selection scheme will be illustrated in the next section.

#### 4.2.2. The Localization Error Analysis of DRSL Algorithm

**Figure 9:**
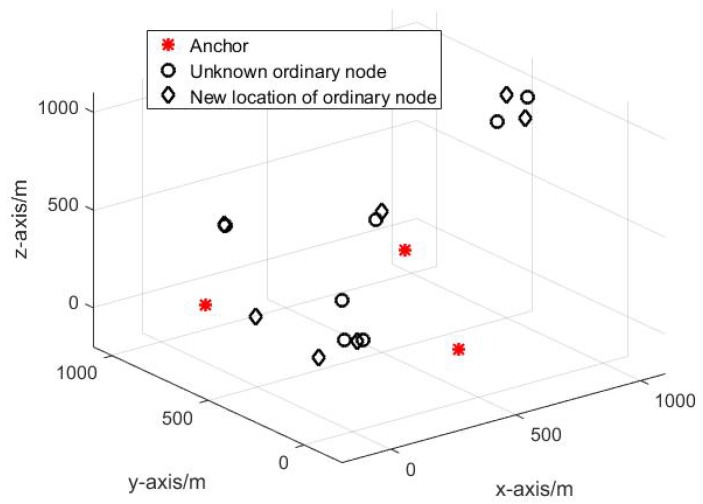
The topology change of DUANs.

As it can be seen in Figure 9, the topology of DUANs is changing overtime. Therefore, the dynamic reference selection-based self-localization algorithm is essential for the mobile network.

The proposed DRSL algorithm is based on a dynamic reference selection scheme and a prediction-based localization method. The reference selection and the localization error described in the last section are set to be the initial value for DRSL algorithm.

**Figure 10:**
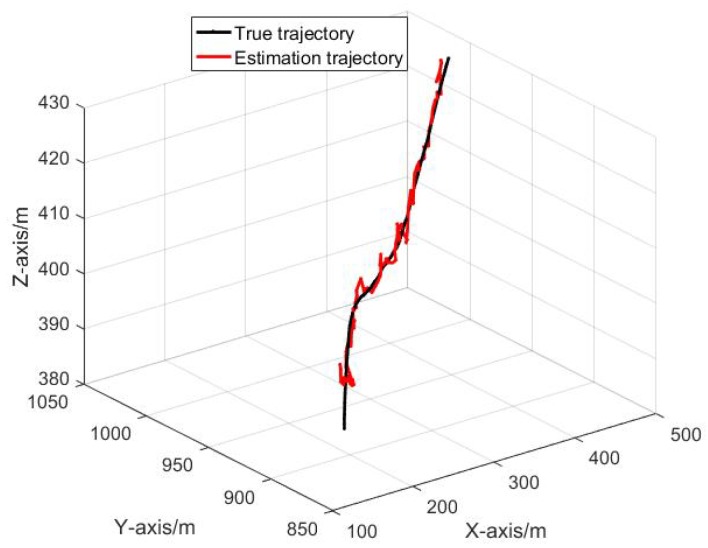
The trajectory of one mobile node in the network.

Figure 10 shows the trajectory of one mobile node in the network.

**Figure 11:**
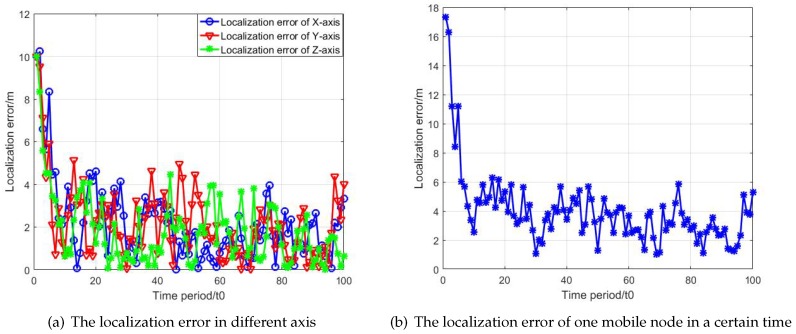
The Localization error analysis.

Figure 11 illustrates the localization error of the mobile node in a certain time. The variance of observation error is 5.

**Figure 12:**
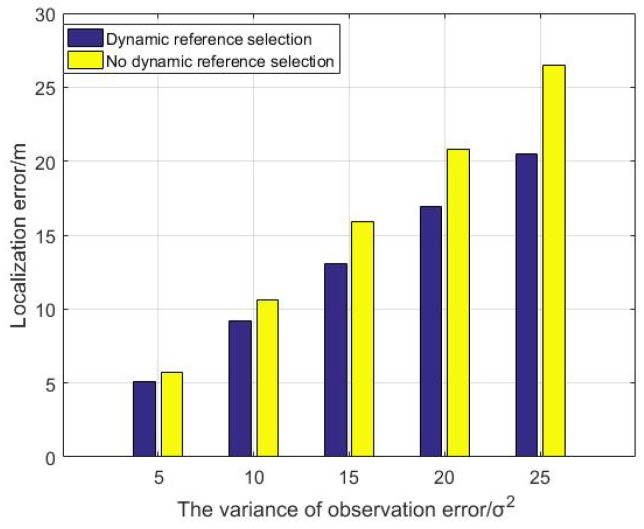
The localization error analysis of dynamic reference selection scheme.

Compare the localization error between the algorithm with dynamic reference selection scheme and the algorithm without dynamic reference selection scheme by doing 500 Monte Carlo experiments in Figure 12. Each experiment has a random and different initial topology.

As can be seen in Figure 12, With different range measurement variance, the localization error of the dynamic reference selection scheme is less than that of the scheme without dynamic reference selection. Figure 12 demonstrates that the dynamic reference selection scheme can greatly improve the localization performance for DUANs with network topology changing problem.

**Figure 13:**
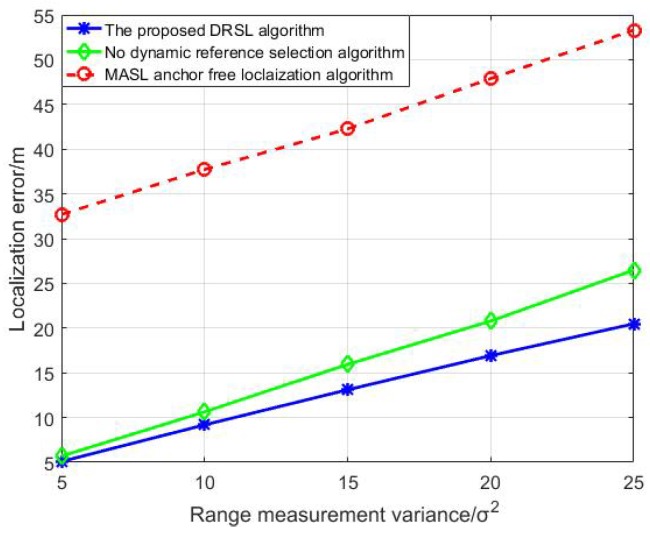
The localization error analysis of different algorithms.

Compare the localization accuracy of the proposed DRSL algorithm with more different algorithms. The result is shown in Figure 13.

As can be seen in Figure 13, the localization error of all these algorithms in Figure 13 increase with the growth of the range measurement variance. Furthermore, the proposed DRSL algorithm has the highest localization precision among the three algorithms. This is because the proposed DRSL algorithm can both assist insufficient anchors in accomplishing the localization procedure with high precision and solve the topology changing problem in DUANs with mobile sensor nodes. The MASL algorithm is an anchor-free algorithm which can work with insufficient anchors; however, it cannot be adapted to the mobile sensor network environment and will be influenced by the low transmission rate in underwater environment dramatically which leads to the highest localization error consequently.

#### 4.2.3. The Communication Cost Analysis

In this section, we compare the proposed DRSL algorithm with anchor-free approach on communication cost. Communication cost is the transmission data size which is needed for the localization procedure. Since nodes in DUANs always have limited power, meanwhile, communication is one of the most important part of power consumption during the localization procedure. Communication cost is an essential performance metric for the localization algorithm.

**Figure 14:**
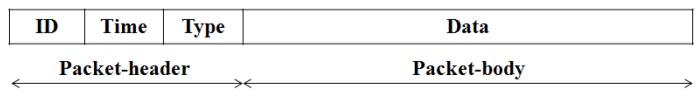
The packet structure.

The sending message in this algorithm usually has two parts: a packet-header and a packet body. We design the packet structure as in Figure 14.

Assume that there are *M* nodes deployed in the network. The time duration of the self-localization procedure is *T*. The test period is $T_{s}$. The estimation period is $T_{0}$

$ID_{s}$ is the node’s ID that sends message, $log_{2}M$ bits;
Time is the time stamp, 2 bytes;
Type is the message’s type, 2 bits

There are 3 types of messages which represent the packet body’s length.

(1) “Hello” packet: “Hello” packet contains the sending anchor’s coordinate. Thus, the length of “Hello” packet is 4 bytes.

(2) “Range” packet: “Range” packet only triggers the range measurement process. Thus, the length of “Range" packet is 0 bit.

(3) “Error” packet: “Error” packet contains the receiving node’s ID and the least square error. Thus, the length of "Error" packet is $log_{2}M+10$ bits.

(4) “Reference” packet: “Reference” packet contains the reference node’s ID and coordinate. Thus, the length of “Reference” packet is $\frac{1}{8}log_{2}M+4$ bytes.

The communication cost *C* is as shown in Equation (15)

(15) $$C=\frac{TT_{0}}{T_{s}}\left[\left(3M-4\right)log_{2}M+\left(2^{10}+14\right)M+\left(2^{9}-1\right)6\right]\;bits$$

**Figure 15:**
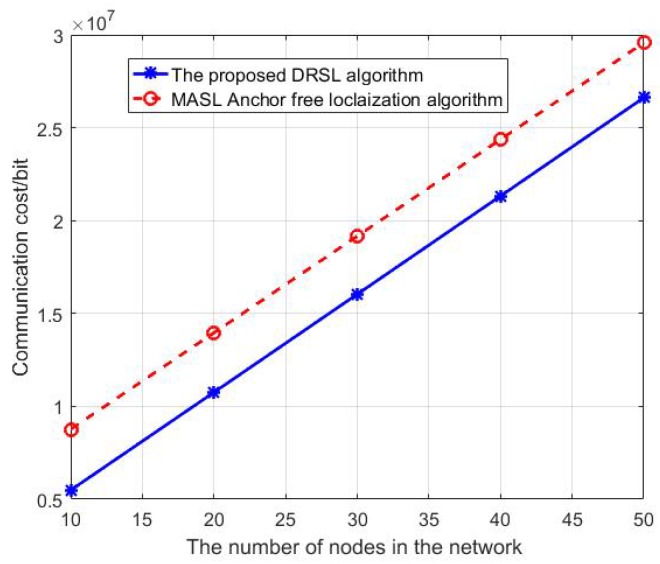
The communication cost analysis.

Increase the number of nodes in the network from 10 to 50. The time duration of localization *T* is set to be $1000t_{0}$ and the estimation period $T_{0}$ is set to be $100t_{0}$ and the test period $T_{s}$ is set to be $200t_{0}$. The comparison between the DRSL algorithm and the MASL anchor-free approach on communication cost is shown in Figure 15.

As shown in Figure 15, the communication cost increases with the number of nodes in the network. This is reasonable since more nodes mean more messages are needed to transmit during the localization procedure. Furthermore, we can see that the communication cost of DRSL algorithm is lower than that of MASL algorithm with no anchor. This is because the proposed DRSL algorithm does not need to do a complete localization process during each time period and does not always have a high sampling frequency which reduce the communication cost of the localization procedure obviously.

## 5. Conclusions

In this paper, we focus on the problem of accurate self-localization for DUANs which has insufficient anchors and varying topology. For this problem, we propose a dynamic reference selection-based self-localization algorithm for DUANs. First, we present an optimal reference selection scheme to solve the insufficient anchors’ problem. The optimal reference node can assist the insufficient anchors in accomplishing the localization procedure and can increase the localization accuracy obviously. Secondly, a dynamic reference selection-based self-localization algorithm is proposed by combining the prediction-based self-localization algorithm with the optimal reference selection scheme. The algorithm can select the reference node dynamically according to the predicted topology and mobility pattern which is more suitable for DUANs with mobile sensor nodes. Simulation results show that the proposed DRSL algorithm can increase the localization accuracy greatly with insufficient anchor nodes and has lower communication cost than other anchor-free approach which demonstrate the advantages of the proposed DRSL algorithm distinctly.
